# A patient decision aid for antidepressant use in pregnancy: study protocol for a randomized controlled trial

**DOI:** 10.1186/s13063-016-1233-4

**Published:** 2016-02-29

**Authors:** Simone Vigod, Neesha Hussain-Shamsy, Sophie Grigoriadis, Louise M. Howard, Kelly Metcalfe, Tim F. Oberlander, Carrie Schram, Donna E. Stewart, Valerie H. Taylor, Cindy-Lee Dennis

**Affiliations:** Women’s College Hospital and Research Institute, 76 Grenville Street, Toronto, ON M5S 1B2 Canada; University of Toronto, 1 King’s College Circle, Toronto, ON M5S 1A8 Canada; Sunnybrook Health Sciences Centre, 2075 Bayview Avenue, Toronto, ON M4N 3M5 Canada; King’s College London, Institute of Psychiatry, Box P031, De Crespigny Park, London, SE5 8AF United Kingdom; Department of Pediatrics, University of British Columbia, Child and Family Research Institute, 4480 Oak St., Vancouver, BC V6H 3V4 Canada; University Health Network, 200 Elizabeth Street, Toronto, ON M5G 2C4 Canada

**Keywords:** Depression, Pregnancy, Women, Patient decision aid, Randomized control trial

## Abstract

**Background:**

Many women with depression experience significant difficulty making a decision about whether or not to use antidepressant medication in pregnancy. Patient decision aids (PDAs) are tools that assist patients in making complex health decisions. PDAs can reduce decision-making difficulty and lead to better treatment outcomes. We describe the methods for a pilot randomized controlled trial of an interactive web-based PDA for women who are having difficulty deciding about antidepressant drug use in pregnancy.

**Methods/Design:**

This is a pilot randomized controlled trial that aims to assess the feasibility of a larger, multi-center efficacy study. The PDA aims to help a woman: (1) understand why an antidepressant is being recommended, (2) be knowledgeable about potential benefits and risks of treatment and non-treatment with antidepressants, and (3) be clear about which benefits and risks are most important to her, with the goal of improving confidence in her decision-making. We include women aged 18 years or older who are: (1) planning a pregnancy or are pregnant (gestational age less than 30 weeks), (2) diagnosed with major depressive disorder, (3) deciding whether or not to use a selective serotonin reuptake inhibitor (SSRI) or serotonin norepinephrine reuptake inhibitor (SNRI) antidepressant in pregnancy, and (4) having at least moderate decision-making difficulty as per a Decisional Conflict Scale (DCS) Score ≥25. Participants are randomized to receive the PDA or an informational resource sheet via a secure website, and have access to the stated allocation until their final study follow-up. The primary outcomes of the pilot study are feasibility of recruitment and retention, acceptability of the intervention, and adherence to the trial protocol. The primary efficacy outcome is DCS score at 4 weeks post randomization, with secondary outcomes including depressive and anxiety symptoms.

**Discussion:**

Our PDA represents a key opportunity to optimize the decision-making process for women around antidepressants in pregnancy, leading to effective decision-making and optimizing improved maternal and child outcomes related to depression in pregnancy. The electronic nature of the PDA will facilitate keeping it up-to-date, and allow for widespread dissemination after efficacy is demonstrated.

**Trial registration:**

This trial is registered on ClinicalTrials.Gov under the identifier NCT02308592 (first registered: 2 December 2014).

**Electronic supplementary material:**

The online version of this article (doi:10.1186/s13063-016-1233-4) contains supplementary material, which is available to authorized users.

## Background

Depression complicates about 10 % of pregnancies around the world [1, 2]. Some women present with a relapse of pre-existing depression and others present with new onset of illness. Untreated or incompletely treated, depression has serious short- and long-term impact on maternal health and exposed children [3, 4]. Depression in pregnancy affects the health and quality of life of the woman, and untreated is associated with increased risk for postpartum depression and chronic maternal depression, conditions linked to impaired mother-infant interactions and poor developmental and emotional outcomes in the offspring [5–12]. Children exposed to depression in utero are at risk for preterm birth, lower birth weight, small head circumference, and lower Apgar scores [3, 4]. Exposure to depression in utero is also associated with childhood problems that extend past the neonatal phase such as delayed infant speech perception [13]. Unfortunately, although depression is one of the most common morbidities in pregnancy, as few as 20 % of women receive treatment [4]. Due to the negative consequences of depression in pregnancy, there is urgency to ensure effective treatment.

There are two standard types of treatment for depression in pregnancy. Psychotherapy is indicated as acute therapy for depression of mild, and sometimes moderate, severity in pregnancy, but psychotherapy alone is unlikely to be effective if a woman has severe depression [14]. Even when psychotherapy is effective, it may take several weeks to months for symptoms to improve, leaving the mother and fetus exposed to the effects of untreated depression during that time. First-line antidepressant medications such as selective serotonin reuptake inhibitors (SSRIs), or serotonin-norepinephrine reuptake inhibitors (SNRIs) are effective for treatment of depression and prevention of relapse [14–20]. Approximately 67 % of individuals achieve remission using antidepressant medication [21]. In addition, as many as 68 % of women who discontinue antidepressant use during pregnancy suffer relapse, leaving themselves and their infants susceptible to the effects of untreated depression [22]. However, the use of antidepressant medication in pregnancy must be considered in the context of the safety of antidepressants for mother and fetus. This is a complex decision because no treatment option is without risk. While depression itself may increase risk for adverse maternal and child outcomes, exposure to antidepressant medication in utero has been associated with small increased risks of neonatal cardiovascular malformations, and neonatal pulmonary hypertension [23–25]. Spontaneous abortions, low birth weight and preterm birth have also been reported in exposed infants, although there is controversy as to whether these risks are higher than among women with untreated depression [26, 27]. Transient short-term adverse neuro-behavioural neonatal effects have been more consistently reported, but only in very rare cases (i.e., less than 1 in 1000) have they been associated with serious effects such as seizures [28–40]. Long-term effects of in-utero exposure are difficult to disentangle from effects of genetics and maternal mood but these drugs cross the placenta and the fetal blood-brain-barrier, suggesting that long-term impact is possible [41, 42]. Many women have significant difficulty making a decision about whether or not to use antidepressant medication in pregnancy. In our previous research, more than 50 % of women faced with this decision displayed high levels of decision conflict, a construct associated with delayed, and ineffective treatment decisions [43, 44]. This supports other research indicating that women report that the complexity of this decision is a barrier to overall treatment uptake for depression in pregnancy [45].

Patient decision aids (PDAs) are evidence-based tools designed to help people engage in choices among options by providing information on the options and outcomes relevant to health status [46]. PDAs are used to supplement, not replace, consultation between health care professionals and patients and aim to prepare individuals to make complex health decisions such as this one where no treatment decision is without risk [47–51]. A Cochrane Collaboration systematic review and meta-analysis of 115 studies demonstrated that patients who use PDAs have better knowledge of their options and more realistic expectations of possible outcomes [47]; and that PDAs can be effective at reducing decisional conflict. We developed a web-based interactive PDA to help women who are pregnant or planning a pregnancy and who are deciding whether or not to use an antidepressant to treat depression. The PDA was developed by a team of perinatal psychiatry experts in Ontario, Canada, in collaboration with front-line perinatal mental health providers, PDA experts and a health care technology company, QoC Health. Herein we present the methodology of a pilot randomized controlled trial (RCT) that evaluates the feasibility of a trial protocol to evaluate the efficacy of our PDA (Research Protocol Version 8; 24 November 2015). The primary objective of the current protocol is to assess feasibility, acceptability and adherence with a prospective, two-armed pilot RCT protocol to evaluate the efficacy of the PDA for women making decisions about the treatment of their depression with an antidepressant during pregnancy. This will guide the development of a larger RCT to definitively evaluate the efficacy of the PDA.

## Methods/Design

### Study design, setting and recruitment

**Fig. 1:**
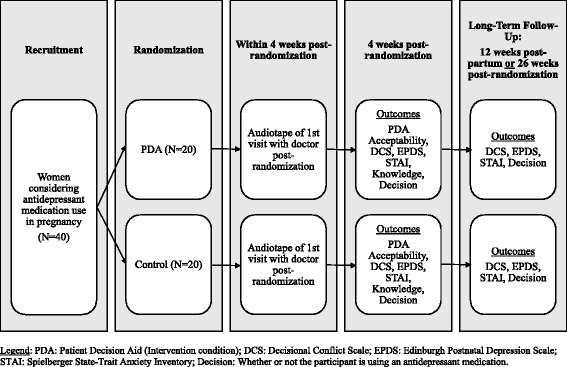
Trial scheme for a pilot randomized control trial for a patient decision aid (PDA) for antidepressant use in pregnancy

This is a two-armed, pilot RCT enrolling women with depression who are pregnant or planning a pregnancy and are having difficulty deciding whether or not they should start or continue an SSRI or SNRI antidepressant for treatment of depression in pregnancy (this is shown in detail in Fig. 1, see Additional file 1). Participants are being recruited over 1 year from a specialty perinatal mental health program at Women’s College Hospital (WCH) in Toronto, Ontario, Canada (population approximately 3.5 million) that receives referrals for mental health care of women during pregnancy from all over the Greater Toronto Area, and the associated family practice program at WCH. Psychiatrists or family physicians at WCH can refer patients directly to the study. To encourage recruitment, referring providers including psychiatrists, family physicians, obstetricians and midwives have been informed of the study; and it has been advertised on WCH social media platforms. The website for the PDA is maintained by a specialty information technology (IT) vendor, QoC Health.

After providing informed consent to the study, participants are assigned a participant identification (ID), affiliated with a randomization allocation, in sequence by a research coordinator who is blind to the allocation. Randomization is stratified by study site (i.e., psychiatry or family practice). A unique website log-in is generated for each woman such that she will be presented with her allocated condition upon log-in. The research coordinator administers a baseline questionnaire and each woman is given her unique website log-in information. Follow-up questionnaires for outcome data are administered at 4 weeks after the baseline assessment for collection of primary outcome data; and again for long-term follow-up at 12 weeks postpartum (for women who enrolled while pregnant) or 6 months after baseline (for women who enrolled while planning a pregnancy). All questionnaires for the study are conducted either by phone or in person, depending on the participant’s preference. Outcome data collectors are blind to study group allocation during the baseline assessment and the follow-up questionnaire. Treating physicians are not directly told of participant allocation, but participants are free and encouraged to discuss it with them. The Research Ethics Board (REB) of WCH in Toronto, Ontario, Canada approved the study (REB# 2014-0050-B) and is notified when changes are made to the study protocol. The study is registered with ClinicalTrials.Gov under the identifier NCT02308592; the trial registration is also updated as appropriate.

### Eligibility criteria

Included participants are: (1) female, (2) aged 18 years or older, (3) planning a pregnancy or less than 30 weeks gestation at enrollment, (4) considering starting or continuing SSRI or SNRI antidepressant medication in pregnancy for major depressive disorder, and (5) have moderate-to-high decision-making difficulty, as defined by a score of ≥25 on the Decisional Conflict Scale [51]. Participants are excluded from the study if they have: (1) had alcohol and/or substance use or dependence in the previous 6 months, (2) active suicidal ideation or psychosis, (3) any major obstetrical complications or fetal cardiac anomalies diagnosed in the current or a past pregnancy (as this changes the risk/benefit ratio discussion in regards to antidepressant use), (4) an inability to read, speak, or understand English and do not have someone that can read the PDA to them, or (5) are otherwise incapable of consenting to participation.

### Informed consent procedures

Potentially eligible women who are willing to hear more about the study receive a detailed study explanation from the study research coordinator. Subjects are provided with a clear explanation of the objectives, procedures, risks and benefits of the study and all questions are answered. Questions are asked of subjects to ensure that they understand the nature of the research, risks and potential benefits of study participation, and their rights as research subjects prior to signing the informed consent document. Interested patients are asked to sign the informed consent form before entry into the study. Informed consent is obtained before any study assessments are performed and before any private information is recorded. Participants are given meaningful opportunities during the study to provide ongoing consent to continuation with the study protocol.

### Allocation of interventions

After informed consent procedures, eligible and consenting women are given a sequential study ID number that is affiliated with a unique username and password for the study website. These ID numbers have been randomized to affiliation with either the intervention or a control condition and are distributed in sequence by a research coordinator who is blind to the allocation. Participants are randomized in a 1:1 ratio with stratification by whether their treating clinician is a psychiatrist or a family physician. When logging into the website using their unique log-in information, participants will see either the PDA or the control condition, a resource sheet with information on depression and antidepressant use in pregnancy.

### Interventions

The PDA is an interactive website that aims to help women: (1) understand why SSRI/SNRI antidepressant medication is being recommended, (2) be knowledgeable about the potential benefits and risks of treatment with antidepressant medication in pregnancy as well as the benefits and risks of not using antidepressant medication, and (3) be clear about which benefits and risks are most important to her, with the goal of improving confidence in her decision-making. To achieve these aims, the PDA intervention has three main sections: (1) evidence-based information about depression in pregnancy and each treatment option and procedure, (2) evidence-based information on the risks and benefits of both untreated depression and antidepressant treatment, integrated with a series of exercises, called “values clarification methods” to help women determine which risks and benefits are most important to them. In keeping with research on the decisional needs of women regarding antidepressant use in pregnancy, it includes explicit exercises to help women consider how their relationships with partners, family, friends, community and providers impact the decision-making process, and (3) a summary section that outlines the information reviewed and which benefits and risks they deemed most important. At the end of the module, participants will see this summary sheet as a printable PDF which can be printed to take with them to their treating clinician for use in clinical follow-up. Women can review the PDA alone or with others if desired (e.g., partner, family, friends). However, the electronic nature of the decision-support tool with secure password also ensures that women can work privately with the PDA. This format also allows women unlimited log-ins so they can complete the PDA at their leisure (or re-read sections/repeat exercises as many times as desired). The intent is to create flexibility in the face of competing demands such as work or childcare. The intervention is written at a grade 6 literacy level and takes approximately 20 minutes to complete. It is best viewed on a desktop or laptop computer. The last page of the PDA is a resource sheet, formatted as a printable PDF with references to standard published information and resources for antidepressant use in pregnancy. Participants who have been allocated the control condition will receive only the resource sheet.

### Study schedule

The study schedule is described in Table 1. A baseline assessment with the research coordinator occurs either in person or by phone, prior to the participant receiving her log-in information for the website. After the baseline assessment, each participant receives her log-in information. Two follow-up questionnaires are then completed in person or by telephone (depending on patient preference): (1) 4 weeks later, and (2) at 12 weeks postpartum for women who enrolled while pregnant, or 6 months post randomization for women who enrolled while planning pregnancy.

**Table 1:** Study schedule for data collection measures and time points

Study variable	Sample	Measure	Baseline	clinic visit	4 weeks	LT F/U^g^
Eligibility	All subjects	Eligibility assessment	Prior to enrollment			
Non-participation		Recruitment issues	Prior to enrollment			
Demographics		Participant characteristics^c^	X			
Mental health		MINI^c, d^	X			
		EPDS^b, e^ and STAI^b, f^	X		X	X
Decisional conflict		Decisional Conflict Scale^b^	X		X	
Knowledge		Knowledge Questionnaire^b^	X		X	
Adherence		Website use and Chart review^a^			X	X
Patient-provider		Audiotaped clinic visit^b^		X		
Decision		Health Service Questionnaire^c^	X		X	X
Participant views	PDA only	Acceptability Questionnaire^a^			X	
Provider views	Providers	Provider Perspective Survey^a^				X^h^

^a^Feasibility; ^b^Efficacy; ^c^Covariate; ^d^Mini Neuropsychiatric Interview; ^e^Edinburgh Postnatal Depression Scale; ^f^State-Trait Anxiety Inventory; ^g^ *LT F/U* = long-term follow-up: at 12 weeks postpartum (for women enrolled while pregnant) or 6 months after baseline assessment (for women enrolled while planning a pregnancy); ^h^Collected only after all other subject data collection is complete. *PDA* patient decision aid

### Outcomes

The primary outcomes for the pilot study are feasibility, acceptability, and adherence with the trial protocol. Feasibility describes how well the trial protocol can be implemented. We record feasibility data related to: (1) eligibility (e.g., the rate of eligible women in each practice), (2) recruitment (e.g., recruitment rate and reasons for non-participation), and (3) timing (e.g., average time from study enrollment to participant use of the PDA). Acceptability refers to women’s satisfaction with and perceptions of the PDA. We record acceptability data on: (1) the participants’ views of the PDA, (2) the rate of completion of the PDA and reasons for discontinuation (e.g., the number of women who view all pages of the PDA), and (3) measures related to participant PDA use (e.g., the length of time it takes to complete the PDA, the number of log-ins required to complete the PDA, average number of times the PDA is completed, and the average number of PDA page views, rate of use of PDA summary sheet in clinical follow-up), and (4) clinical providers’ views of the PDA. Lastly, we assess adherence, defined as the degree to which the trial protocol is followed. Adherence-related measures include: (1) the number of women who see their clinical provider for follow-up within 4 weeks of enrolling in the study, and (2) the follow-up rate for data collection.

Secondary outcomes for the pilot study are the efficacy of the PDA as an adjunct to clinical care on decisional conflict (primary outcome for the future efficacy RCT), psychiatric symptoms, and knowledge about depression and antidepressant use in pregnancy. Provider views are also collected. Decisional conflict is measured using the Decisional Conflict Scale (DCS). The purpose of this scale is to measure a person’s perception of difficulty in making a decision including: (1) uncertainty in choosing between options, (2) modifiable factors contributing to uncertainty such as feeling uninformed, being unclear about personal values, and unsupported, and (3) quality of the choice selected, which is defined as informed and consistent with personal values, and with which a person expresses personal satisfaction [43]. The DCS consists of 16 items (ranging from 1 to 5, with 5 being high decisional conflict). Scores of 25 or greater are associated with those who delay decisions. Test-retest and internal consistency coefficients exceed 0.78. Depressive and anxious symptoms are measured using the Edinburgh Postnatal Depression Scale (EPDS) and the State-Trait Anxiety Inventory (STAI) respectively. The EPDS is a self-report depression screening measure that has been validated for use in pregnancy [52]. The EPDS has a positive predictive value of 73 % at a cut-off score of 12/13 [53]. The STAI is a self-report screening measure for anxiety that has shown good discriminately validity in perinatal populations. STAI scores >48 are predictive of having an anxiety disorder diagnosis [54]. Knowledge of depression treatment options is assessed using a knowledge questionnaire that includes items regarding the effectiveness of various treatment options, as well as known possible adverse effects. Women will be asked to estimate effectiveness of each presented treatment option on a continuous scale from 0 to 100 %. Content knowledge items are presented as “true” or “false.” This tool is a modified version of a tool previously created and validated by a member of our team in other PDA evaluations (KM) [55]. Provider views about their perceptions of the utility of the PDA for their patients are also collected using a modified version of a provider satisfaction tool for PDAs created for previous studies by members of our team (KM) [55].

We also audio-record the first clinical interaction between each patient and her provider subsequent to randomization (see Table 1). Audiotapes will be transcribed verbatim and analyzed using qualitative methods. This method was developed in conjunction with the teams of family physicians and psychiatrists who will be seeing the study patients. They will have the opportunity to review the PDA and the research protocol prior to the start of the study and can opt out of the audiotaping at any time. Providers will provide clinical care to study participants in both groups. If providers learn from interactions with women in the intervention (PDA) group, this could change interactions with all patients. If this positively impacts decisional conflict for participants in the control group, the observed effect size of the PDA intervention could be reduced [56]. While cluster randomization (e.g., by site or by provider) could minimize such a bias, this raises issues of feasibility, size, cost, as well as issues of systematic differences between study sites (or between providers) that could confound results. As such, the impact of provider learning needs to be considered to inform the design of the future RCT. To assess for a possible impact of provider learning, we will analyze the temporal trends in decisional conflict scale outcomes over time in both groups, and compare temporal trends in decisional conflict scale outcomes between PDA and control groups. If decisional conflict outcomes in the control group are better for participants enrolled later in the study, compared to participants enrolled in the early part of the study, a cluster-randomized design may be preferred in the future RCT. Similar methods have been used to assess the impact of provider learning over time in interventions involving the use of new surgical skills and procedures [57].

### Statistical methods

Means will be calculated to determine feasibility and compliance estimates for recruitment rate, rates of non-participation, completion rate of the PDA, number of pages reviewed within active study phase, follow-up rates at the various intervals, and so on. We will measure the acceptability of the intervention using the Likert-type scale responses from the participant and provider questionnaires, and will collate additional comments made by participants or providers for consideration of modifications to the content, layout or technical capacity of the PDA. To compare decisional conflict between women receiving the PDA to women receiving the comparison condition we will use the intention-to-treat principle. Missing data points will be excluded from the analysis, and individuals with less than two DCS measurements will be treated as lost to follow-up. An on-treatment analysis will also be performed as a sensitivity analysis (for best possible performance of the PDA). Means of the DCS scores of the experimental and comparison groups at the primary endpoint (4-week follow-up) will be compared using a one-way analysis of covariance (ANCOVA) model, where the covariate will be baseline DCS score. This effect size will be used to generate the sample size needed for the future RCT. For other secondary outcomes, continuous measures will be compared between groups using *t* tests for independent samples (or ANCOVA to account for baseline scores) and dichotomous measures using chi-square tests of association. Audiotaped participant-provider visits will be transcribed verbatim and analyzed using inductive thematic qualitative content analysis. The frequency, extensiveness and specificity of comments will guide data categorization into recurrent themes. Themes will be altered and refined through a recursive process from the data to analyst-generated categorical and conceptual definitions. NVivo software will be used to facilitate comparisons between experimental and comparison groups. Recruitment will continue for 1 year, and no analysis performed until the last participant has completed final follow-up.

### Sample size

A review by Hertzog suggests a range of 20–40 participants to allow for sufficient variability in acceptability assessment of an intervention [58]. The RLS program at WCH is a specialty program that treats women experiencing mental health concerns related to the reproductive life stages (including menstruation, pregnancy, postpartum and menopause) and conducts approximately 40 consultations per month for perinatal depression; of these approximately 11 have moderate to severe depression in pregnancy. Based on a previous study on decisional conflict [44], we estimate that six (approximately 50 %) will be ineligible due to low decisional conflict, one will be ineligible for another reason and three of the remaining five (60 %) will agree to participate in the study. As such, recruitment of 36 women could be achieved through this clinic in 1 year. WCH also has a Family Practice clinic that cares for approximately 350 pregnant women per year. Estimating an eligibility rate of 11 %, 39 would be eligible for the study, so 24 (60 %) women could be recruited by this site over a 1-year period. In total, this would allow for recruitment of 50 participants over the course of 1 year. We aim to recruit at least 25 participants to the intervention group, allowing for 20 % loss to follow-up for a minimum of 20 per group. Among women with mean DCS score = 30 (moderate-high) and SD 14.8 (based on pilot data), a total sample size of 40 participants would allow us to detect a 13-point difference in DCS scores (clinically significant) between intervention and control groups with probability (power) 0.8 (type 1 error rate or alpha = 0.05).

### Safety monitoring

A committee consisting of the principal investigator (PI), study co-investigators and the research assistant will hold teleconferences as necessary to discuss study progress, including participant recruitment, and unexpected issues. Because of the low-risk nature of this intervention, an independent Data Safety Monitoring Board will not be developed. Safety will be assessed at all follow-up time points and be routinely reviewed by the PI. Adverse events will be recorded and serious adverse events immediately reported (within 24 hours by telephone or fax) to the WCH REB for consideration of further action (i.e., unblinding of intervention, subject withdrawal, termination of study). Protocols will be developed regarding specific criteria for termination during the active study phase that will include active suicidal ideation, psychosis and acute pregnancy complications that might change the considerations required for treatment decisions. If termination criteria are met, the participant will be removed from the study and followed by her physician. Participants who terminate early will form part of the intention-to-treat analysis.

### Confidentiality

Participant confidentiality will be maintained by using an ID number (not related to name or date of birth) on all documents. Information linking these ID numbers with the subjects’ identity will be kept separate from the research records. Computer-based data will be entered into password-secured databases and paper files stored in a secure location. Data will only be accessible to study personnel. User profile and system data stored and transmitted by the study website is secured both technically and by business practices in compliance with Government of Canada privacy standards. Data will be hosted entirely within Canada adhering to jurisdictional compliance standards. Data will be stored after the end of the study on a password-protected electronic archive for the PI to access for 5 years. The approving REB will be granted direct access to the study participants’ original medical records for verification of trial procedures and/or data, without violating the confidentiality of the participants, to the extent permitted by law and regulations. In presenting results, participants’ identities will be confidential.

### Dissemination plan

The goal of this pilot study is to finalize the development of a PDA for antidepressant use in pregnancy and to determine whether it is feasible to conduct a larger RCT to definitively evaluate the PDA. The goals of the KT plan relevant to this are to: (1) inform future research in terms of providing evidence to proceed to a larger study of the efficacy of the PDA, and (2) generate awareness and interest in both the research community and in the public about the potential of the PDA. To meet the first goal of the KT plan, potential partners for a planned larger trial require engagement. Our multi-disciplinary team has connections to centers throughout Canada, the United States and the UK, and pending success of the pilot study we will hold a trial-planning meeting after data collection is complete to prepare for a larger multi-site RCT to definitively evaluate the PDA. To meet the second goal of the KT plan, practitioners, the public, stakeholder groups and policy-makers are target audiences. Specifically, the team has ongoing relationships with several community and public health agencies and stakeholder groups that focus on perinatal depression. We will share pilot study findings with these key stakeholders, many of whom were instrumental in the PDA development to date. These presentations are integrated knowledge translation activities as they are bi-directional in nature. Stakeholders will help inform us, while at the same time serving to disseminate information and generate interest and awareness about the potential of the PDA as an intervention. Finally, to promote the legitimacy of our findings, we will submit results for peer-reviewed publication and present the results of the pilot study (once peer-reviewed) in at least one national and international scientific conference.

## Discussion

Depression complicates a large number of pregnancies, more often than gestational diabetes, hypertension or pre-eclampsia [1]. Furthermore, depression in pregnancy poses a significant risk to both mother and developing fetus, yet few women receive adequate care. Antidepressant medication is often the most effective treatment option, but is associated with risks. This causes women to struggle with decisions around their treatment options, despite physician and informational support. PDAs are interventions that reduce decisional conflict and enhance effective decision-making. To our knowledge, there has been no other PDA for antidepressant use in pregnancy that has been rigorously evaluated for efficacy as an adjunct to clinical care.

The electronic nature of our tool is a major advantage to our protocol and its scalability, because it means that: (1) women will be able to access it privately and securely, augmenting care particularly in areas where access to specialists is scarce, (2) it will be feasible to update the contained information on a regular basis to keep up with the consistently changing literature in this area, (3) it is easily adaptable and disseminated to other settings. For example, we have developed a collaboration with King’s College in the UK where the PDA is being adapted to suit the context of mental health care in the UK (i.e., different brand names of medication, etc.) and where a pilot study in the Greater London Area has been funded to pilot test the PDA in that setting.

There are some limitations to our pilot study methodology. We are restricting our sample to women who are less than 30 weeks gestation to increase the likelihood that the decision would be implemented during the pregnancy. The disadvantage of this is that our results may not be applicable to women in late pregnancy. Also, because this is a pilot study, our results will not provide us with conclusions about the efficacy of the PDA for women making decisions about antidepressant use in pregnancy, but rather, will help guide the development of a larger study to focus on this matter.

Deciding whether or not to treat depression with antidepressants during pregnancy can be a complex decision for women to make, even with clinical care and guidance. Our PDA recognizes that women must be able to assess her own values of the risks and benefits of her treatment options in order to make an informed and effective decision. This study will allow us to determine the feasibility of our protocol for a larger, multi-site RCT in order to determine the efficacy of a relatively simple, low-cost intervention that has the potential to help a significant number of women and unborn children.

### Trial status

Enrollment for this study began 1 February 2015. At the time of article submission we have enrolled 37 participants.

## Additional file

Additional file 1: SPIRIT 2013 Checklist: recommended items to address in a clinical trial protocol and related documents* (DOCX 48 kb)

## Abbreviations

ANCOVA: analysis of covariance
DCS: Decisional Conflict Scale
EPDS: Edinburgh Postnatal Depression Scale
PDA: patient decision aid
RCT: randomized control trial
REB: Research Ethics Board
STAI: State-Trait Anxiety Inventory.

## References

[1] Bennett HA (2004). Prevalence of depression during pregnancy: systematic review. Obstet Gynecol.

[2] Gavin NI (2005). Perinatal depression: a systematic review of prevalence and incidence. Obstet Gynecol.

[3] Wisner KL (2009). Major depression and antidepressant treatment: impact on pregnancy and neonatal outcomes. Am J Psychiatry.

[4] Marcus SM (2009). Depression during pregnancy: rates, risks and consequences – Motherisk Update 2008. Can J Clin Pharmacol.

[5] Beck CT (1998). The effects of postpartum depression on child development: a meta-analysis. Arch Psychiatr Nurs.

[6] Hammen C, Brennan PA (2003). Severity, chronicity, and timing of maternal depression and risk for adolescent offspring diagnoses in a community sample. Arch Gen Psychiatry.

[7] Martins C, Gaffan EA (2000). Effects of early maternal depression on patterns of infant-mother attachment: a meta-analytic investigation. J Child Psychol Psychiatry.

[8] Murray L (1992). The impact of postnatal depression on infant development. J Child Psychol Psychiatry.

[9] Nulman I (2002). Child development following exposure to tricyclic antidepressants or fluoxetine throughout fetal life: a prospective, controlled study. Am J Psychiatry.

[10] O’Hara MW, Swain AM (1996). Rates and risk of postpartum depression – A meta-analysis. Int Rev Psychiatry.

[11] Sinclair D, Murray L (1998). Effects of postnatal depression on children’s adjustment to school. Teacher’s reports. Br J Psychiatry..

[12] Weinberg MK, Tronick EZ (1998). The impact of maternal psychiatric illness on infant development. J Clin Psychiatry..

[13] Weikum WM (2012). Prenatal exposure to antidepressants and depressed maternal mood alter trajectory of infant speech perception. Proc Natl Acad Sci U S A..

[14] CANMAT (2001). Clinical guidelines for the treatment of depressive disorders. Can J Psychiatry..

[15] Dennis CL (2004). Treatment of postpartum depression, part 2: a critical review of nonbiological interventions. J Clin Psychiatry.

[16] Dennis CL (2004). Preventing postpartum depression part I: a review of biological interventions. Can J Psychiatry.

[17] Dennis CL (2004). Preventing postpartum depression part II: a critical review of nonbiological interventions. Can J Psychiatry.

[18] Dennis CL, Stewart DE (2004). Treatment of postpartum depression, part 1: a critical review of biological interventions. J Clin Psychiatry.

[19] Gjerdingen D (2003). The effectiveness of various postpartum depression treatments and the impact of antidepressant drugs on nursing infants. J Am Board Fam Pract.

[20] Sharma V (2002). Pharmacotherapy of postpartum depression. Expert Opin Pharmacother.

[21] Rush AJ (2006). Acute and longer-term outcomes in depressed outpatients requiring one or several treatment steps: a STAR*D report. Am J Psychiatry.

[22] Cohen LS (2006). Relapse of major depression during pregnancy in women who maintain or discontinue antidepressant treatment. JAMA.

[23] Pedersen LH (2009). Selective serotonin reuptake inhibitors in pregnancy and congenital malformations: population based cohort study. BMJ..

[24] Chambers CD (2006). Selective serotonin-reuptake inhibitors and risk of persistent pulmonary hypertension of the newborn. N Engl J Med.

[25] Kieler H (2012). Selective serotonin reuptake inhibitors during pregnancy and risk of persistent pulmonary hypertension in the newborn: population based cohort study from the five Nordic countries. BMJ..

[26] Wen SW (2006). Selective serotonin reuptake inhibitors and adverse pregnancy outcomes. Am J Obstet Gynecol.

[27] Wisner KL (2013). Does fetal exposure to SSRIs or maternal depression impact infant growth?. Am J Psychiatry.

[28] Casper RC (2003). Follow-up of children of depressed mothers exposed or not exposed to antidepressant drugs during pregnancy. J Pediatr.

[29] Chambers CD (1996). Birth outcomes in pregnant women taking fluoxetine. N Engl J Med.

[30] Cohen LS (2000). Birth outcomes following prenatal exposure to fluoxetine. Biol Psychiatry.

[31] Costei AM (2002). Perinatal outcome following third trimester exposure to paroxetine. Arch Pediatr Adolesc Med.

[32] Ericson A, Kallen B, Wiholm B (1999). Delivery outcome after the use of antidepressants in early pregnancy. Eur J Clin Pharmacol.

[33] Kulin NA (1998). Pregnancy outcome following maternal use of the new selective serotonin reuptake inhibitors: a prospective controlled multicenter study. JAMA.

[34] Laine K (2003). Effects of exposure to selective serotonin reuptake inhibitors during pregnancy on serotonergic symptoms in newborns and cord blood monoamine and prolactin concentrations. Arch Gen Psychiatry.

[35] Levinson-Castiel R (2006). Neonatal abstinence syndrome after in utero exposure to selective serotonin reuptake inhibitors in term infants. Arch Pediatr Adolesc Med.

[36] Nordeng H (2001). Neonatal withdrawal syndrome after in utero exposure to selective serotonin reuptake inhibitors. Acta Paediatr.

[37] Oberlander TF (2002). Prolonged prenatal psychotropic medication exposure alters neonatal acute pain response. Pediatr Res.

[38] Pastuszak A (1993). Pregnancy outcome following first-trimester exposure to fluoxetine (Prozac). JAMA.

[39] Simon GE, Cunningham ML, Davis RL (2002). Outcomes of prenatal antidepressant exposure. Am J Psychiatry.

[40] Stiskal JA (2001). Neonatal paroxetine withdrawal syndrome. Arch Dis Child Fetal Neonatal Ed.

[41] Nulman I (2012). Neurodevelopment of children following prenatal exposure to venlafaxine, selective serotonin reuptake inhibitors, or untreated maternal depression. Am J Psychiatry.

[42] Mulder EJ (2011). Selective serotonin reuptake inhibitors affect neurobehavioral development in the human fetus. Neuropsychopharmacology.

[43] O’Connor AM (1995). Validation of a decisional conflict scale. Med Decis Making.

[44] Walton GD, Ross LE, Stewart DE, Grigoriadis S, Dennis CL, Vigod S. Decisional conflict among women considering antidepressant medication use in pregnancy. Arch Womens Ment Health. 2014;17(6):493–501. doi: 10.1007/s00737-014-0448-1. 10.1007/s00737-014-0448-1 25104244

[45] Battle CL (2013). Perinatal antidepressant use: understanding women’s preferences and concerns. J Psychiatr Pract.

[46] Coulter A, Kryworuchko J, Mullen PD, Ng CJ, Stilwell D, van der Weijden T. Chapter A: using a systematic development process, in update of the International Patient Decision Aids Standards (IPDAS) Collaborations’ Background Document, Llewellyn-Thomas HA, Volk RJ, editors. 2012. pp. 1–16. http://ipdas.ohri.ca/resources.html. Accessed 23 Feb 2016.

[47] Stacey D (2011). Decision aids for people facing health treatment or screening decisions. Cochrane Database Syst Rev..

[48] Kasper JF, Mulley AG, Wennberg JE (1992). Developing shared decision-making programs to improve the quality of health care. QRB Qual Rev Bull.

[49] Levine MN (1992). A bedside decision instrument to elicit a patient’s preference concerning adjuvant chemotherapy for breast cancer. Ann Intern Med.

[50] O’Connor AM, Jacobsen MJ, Stacey D (2002). An evidence-based approach to managing women’s decisional conflict. J Obstet Gynecol Neonatal Nurs.

[51] O’Connor AM (1999). The Ottawa patient decision aids. Eff Clin Pract.

[52] Gibson J (2009). A systematic review of studies validating the Edinburgh Postnatal Depression Scale in antepartum and postpartum women. Acta Psychiatr Scand.

[53] Cox JL, Holden JM, Sagovsky R (1987). Detection of postnatal depression. Development of the 10-item Edinburgh Postnatal Depression Scale. Br J Psychiatry.

[54] Dennis CL, Coghlan M, Vigod S. Can we identify mothers at-risk for postpartum anxiety in the immediate postpartum period using the State-Trait Anxiety Inventory? J Affect Disord. 2013;150(3):1217–20. doi: 10.1016/j.jad.2013.05.049. 10.1016/j.jad.2013.05.049 23764383

[55] Metcalfe KA (2007). Development and testing of a decision aid for breast cancer prevention for women with a BRCA1 or BRCA2 mutation. Clin Genet.

[56] Watson M (2005). Measuring contamination in an injury prevention randomized controlled trial. Int J Inj Contr Saf Promot.

[57] Cook JA, Ramsay CR, Fayers P (2004). Statistical evaluation of learning curve effects in surgical trials. Clin Trials.

[58] Hertzog MA (2008). Considerations in determining sample size for pilot studies. Res Nurs Health.

